# Joint Imaging Platform for Federated Clinical Data Analytics

**DOI:** 10.1200/CCI.20.00045

**Published:** 2020-11-09

**Authors:** Jonas Scherer, Marco Nolden, Jens Kleesiek, Jasmin Metzger, Klaus Kades, Verena Schneider, Michael Bach, Oliver Sedlaczek, Andreas M. Bucher, Thomas J. Vogl, Frank Grünwald, Jens-Peter Kühn, Ralf-Thorsten Hoffmann, Jörg Kotzerke, Oliver Bethge, Lars Schimmöller, Gerald Antoch, Hans-Wilhelm Müller, Andreas Daul, Konstantin Nikolaou, Christian la Fougère, Wolfgang G. Kunz, Michael Ingrisch, Balthasar Schachtner, Jens Ricke, Peter Bartenstein, Felix Nensa, Alexander Radbruch, Lale Umutlu, Michael Forsting, Robert Seifert, Ken Herrmann, Philipp Mayer, Hans-Ulrich Kauczor, Tobias Penzkofer, Bernd Hamm, Winfried Brenner, Roman Kloeckner, Christoph Düber, Mathias Schreckenberger, Rickmer Braren, Georgios Kaissis, Marcus Makowski, Matthias Eiber, Andrei Gafita, Rupert Trager, Wolfgang A. Weber, Jakob Neubauer, Marco Reisert, Michael Bock, Fabian Bamberg, Jürgen Hennig, Philipp Tobias Meyer, Juri Ruf, Uwe Haberkorn, Stefan O. Schoenberg, Tristan Kuder, Peter Neher, Ralf Floca, Heinz-Peter Schlemmer, Klaus Maier-Hein

**Affiliations:** ^1^ Division of Medical Image Computing, German Cancer Research Center, Heidelberg, Germany; ^2^ Medical Faculty Heidelberg, University of Heidelberg, Heidelberg, Germany; ^3^ German Cancer Consortium, Heidelberg, Germany; ^4^ Pattern Analysis and Learning Group, Radio-oncology and Clinical Radiotherapy, Heidelberg University Hospital, Heidelberg, Germany; ^5^ Division of Radiology, German Cancer Research Center, Heidelberg, Germany; ^6^ Klinik Diagnostische und Interventionelle Radiologie der Universität Heidelberg, Heidelberg, Germany; ^7^ Institut für Diagnostische und Interventionelle Radiologie, Universitätsklinikum Frankfurt, Frankfurt, Germany; ^8^ Klinik für Nuklearmedizin, Universitätsklinikum Frankfurt, Frankfurt, Germany; ^9^ Institut und Poliklinik für Diagnostische und Interventionelle Radiologie, Universitätsklinikum Carl Gustav Carus Dresden, Dresden, Germany; ^10^ Klinik und Poliklinik für Nuklearmedizin, Universitätsklinikum Carl Gustav Carus Dresden, Dresden, Germany; ^11^ Medical Faculty, Department of Diagnostic and Interventional Radiology, University Düsseldorf, Düsseldorf, Germany; ^12^ Klinik für Nuklearmedizin, Universitätsklinikum Düsseldorf, Düsseldorf, Germany; ^13^ Klinik für Diagnostische und Interventionelle Radiologie, Universitätsklinikum Tübingen, Tübingen, Germany; ^14^ Klinik für Nuklearmedizin und Klinische Molekulare Bildgebung, Universitätsklinikum Tübingen, Tübingen, Germany; ^15^ Department of Radiology, University Hospital, Ludwig Maximilian University Munich, Munich, Germany; ^16^ German Center of Lung Research, Giessen, Germany; ^17^ Klinik und Poliklinik für Nuklearmedizin, Klinikum der Universität München, München, Germany; ^18^ Institut für Diagnostische und Interventionelle Radiologie und Neuroradiologie, Universitätsklinikum Essen AöR, Essen, Germany; ^19^ Klinik für Nuklearmedizin, Universitätsklinikum Essen AöR, Essen, Germany; ^20^ Klinik für Radiologie (mit dem Bereich Kinderradiologie), Charité Universitätsmedizin Berlin, Berlin, Germany; ^21^ Klinik für Nuklearmedizin, Charité–Universitätsmedizin Berlin, Berlin, Germany; ^22^ Klinik und Poliklinik für Diagnostische und Interventionelle Radiologie, Universitätsmedizin Mainz, Mainz, Germany; ^23^ Klinik und Poliklinik für Nuklearmedizin, Universitätsmedizin Mainz, Mainz, Germany; ^24^ Institut für Diagnostische und Interventionelle Radiologie, Klinikum Rechts der Isar, Technical University of Munich, Munich, Germany; ^25^ Department of Computing, Imperial College London, London, United Kingdom; ^26^ Klinik und Poliklinik für Nuklearmedizin, Klinikum Rechts der Isar, Technical University of Munich, Munich, Germany; ^27^ Klinik für Diagnostische und Interventionelle Radiologie, Universitätsklinikum Freiburg, Freiburg, Germany; ^28^ Klinik für Nuklearmedizin, Universitätsklinikum Freiburg, Freiburg, Germany; ^29^ Klinische Kooperationseinheit Nuklearmedizin, Deutsches Krebsforschungszentrum Heidelberg, Heidelberg, Germany; ^30^ Universitätsmedizin Mannheim, Medizinische Fakultät Mannheim der Universität Heidelberg, Heidelberg, Germany; ^31^ Medizinische Physik in der Radiologie, Deutsches Krebsforschungszentrum Heidelberg, Heidelberg, Germany

## Abstract

**PURPOSE:**

Image analysis is one of the most promising applications of artificial intelligence (AI) in health care, potentially improving prediction, diagnosis, and treatment of diseases. Although scientific advances in this area critically depend on the accessibility of large-volume and high-quality data, sharing data between institutions faces various ethical and legal constraints as well as organizational and technical obstacles.

**METHODS:**

The Joint Imaging Platform (JIP) of the German Cancer Consortium (DKTK) addresses these issues by providing federated data analysis technology in a secure and compliant way. Using the JIP, medical image data remain in the originator institutions, but analysis and AI algorithms are shared and jointly used. Common standards and interfaces to local systems ensure permanent data sovereignty of participating institutions.

**RESULTS:**

The JIP is established in the radiology and nuclear medicine departments of 10 university hospitals in Germany (DKTK partner sites). In multiple complementary use cases, we show that the platform fulfills all relevant requirements to serve as a foundation for multicenter medical imaging trials and research on large cohorts, including the harmonization and integration of data, interactive analysis, automatic analysis, federated machine learning, and extensibility and maintenance processes, which are elementary for the sustainability of such a platform.

**CONCLUSION:**

The results demonstrate the feasibility of using the JIP as a federated data analytics platform in heterogeneous clinical information technology and software landscapes, solving an important bottleneck for the application of AI to large-scale clinical imaging data.

## INTRODUCTION

Medical imaging plays an essential role in nearly all aspects of high-quality cancer care, from preventive measures, including screening and early detection, through diagnosis, treatment planning and monitoring, and follow-up. Most patients with cancer undergo repeated imaging during the course of their treatment. In combination with clinical and laboratory data, quantitative imaging biomarkers are of fundamental importance to improve standardized therapy monitoring in clinical multicenter studies.^1-7^

**CONTEXT**

**Key Objective**
To create a digital infrastructure for federated artificial intelligence (AI)–based medical image analysis with the goal of facilitating and enabling multicenter trials between the partner sites of the German Cancer Consortium and beyond.
**Knowledge Generated**
The decentralized local execution of data analyses can solve many obstacles of cross-site collaboration. This work tackles organizational, legal, and technical challenges of distributed data analysis and shows its value in several use cases and studies.
**Relevance**
Translating new developments into clinical practice is the ultimate goal of medical imaging research, and using these new technologies will yield enormous benefits for patients. The open-source Joint Imaging Platform presented in this work is realizing this step from the research laboratory into a real multicenter clinical study setting, supporting and enabling the translation of cutting-edge AI-based technologies into clinical practice.

Medical images are more than pictures; they are data characterizing the patient.^8^ As such, they are rightly subject to strict data protection, as well as ethical and moral requirements for scientific secondary use, which, in turn, can impede the exchange of biomedical imaging data across clinical sites. Current research strongly indicates that data anonymization is not only difficult to perform but also generally ineffective in practice.^9-11^ De-identification or anonymization methods considered safe today might potentially fail in the future.^9^ Data ownership, insufficient personal incentives for data collectors to share their data, and remaining technical challenges present further hurdles to data sharing in the medical research domain.^12^ In addition, the clinical landscape is composed of heterogeneous information technology (IT) systems as well as different scanners and acquisition parameters, making joint projects and data sharing cumbersome but also necessary for generalizable image-based biomarkers and artificial intelligence (AI)–based image analysis.

Recent improvements in machine learning (ML) have enabled algorithms that can achieve results for specialized tasks equivalent to those of physicians and that are able to support human experts in improving their performance and efficiency.^4,13-27^ These accomplishments elevated medical imaging to one of the most promising fields for practical application of ML in health care, aiming at better prediction, diagnosis, and treatment of diseases.^13^ However, to enable a broad application of these techniques, a number of challenges still need to be overcome. The common denominator of all previous success stories is an extensive investment in collecting and curating a substantial amount of multicentric imaging data, which is critical to establish the required robustness of ML models. Thus, the obstacles for data sharing represent a bottleneck for medical research in general and for cancer research in particular.

To overcome this bottleneck, several projects and initiatives are working on facilitating, accelerating, and promoting collaboration in larger scientific networks. Existing platforms such as KETOS,^28^ which is based on DataSHIELD^29,30^ and targeted to perform statistical analysis on textual clinical information, were among the first to adopt the concept of federated on-site execution of algorithms to enable decentralized analysis of clinical data. The field of bioinformatics has brought up widely used platforms for standardized data analysis. Among these, Galaxy^31^ is the most prominent solution for platform-based genome analyses. The Personal Health Train,^32^ on the other hand, envisions federated scenarios that include the continuous federated training of ML models. In the area of medical imaging, Sharma et al^33^ presented the Platform for Imaging in Precision Medicine (PRISM). PRISM focuses on the curation, management, and exploration of radiologic, pathologic, and clinical data collections, such as The Cancer Imaging Archive,^34^ not on the decentralized processing of imaging data and the realization of multicenter trials.

To fill this gap, the strategic initiative Joint Imaging Platform (JIP) was established by the German Cancer Consortium (DKTK).^35^ The DKTK is a long-term initiative by the German Federal Ministry of Education and Research connecting more than 20 academic research institutes and university hospitals with the German Cancer Research Center to foster multicenter clinical trials for improved cancer diagnosis and treatment (Appendix Table A1). The consortium provides a unique opportunity for collecting large-scale high-quality imaging data from several institutions. The JIP is designed to facilitate collaborative imaging projects across institutions by addressing the typical technical, organizational, and legal challenges associated with the sharing of imaging data, acquisition parameters, analysis algorithms, or processing results. By enabling training, evaluation, and application of algorithms in large-scale federated clinical settings, the platform builds a solid and extensible foundation for federated learning scenarios. Leveraging open-source technologies, the JIP has the potential to serve as a promoter of prospective cross-center radiologic studies at unprecedented cohort sizes, not only within the DKTK but also beyond.

## METHODS

The JIP is designed as a federated data analysis and processing system (ie, for delivering methods and tools to the image data instead of collecting the data for processing and analysis). Strict on-site data processing mitigates common problems with data protection regulations because no personal data have to leave the clinic.

### Platform Requirements

We conducted a requirement analysis at each DKTK site to gather information regarding their specific demands and expectations. The collected responses revealed a quite heterogeneous landscape considering patient count, modalities, and IT systems. Furthermore, the analysis revealed two main requirements for the JIP. First, enabling and supporting multicenter imaging studies was of utmost importance, including access to larger case numbers for retrospective data analyses and the facilitation of collaborations. Second, an improved integration of data processing, annotation, and sharing tools into the clinical environment was of interest, particularly ML and federated data analytics. These results were translated into the following individual aspects that should be realized in the platform.

#### Integrability.

The fundamental principle of the JIP is based on the local execution of analysis methods as an extension of the existing clinical infrastructure. As a result, the platform should seamlessly integrate with existing local clinical systems, and the interaction of physicians with the JIP should be as close as possible to the established clinical workflows and tools.

#### Data accessibility.

To achieve high compatibility with existing hospital systems, the widely established standard for Digital Imaging and Communications in Medicine (DICOM) should be used whenever possible. It should also be possible to view stored images and segmentations within the platform. Results of computations (eg, segmentations or parametric images) should be stored in DICOM to ensure high compatibility.

#### Algorithmic accessibility.

The platform should facilitate the sharing and distribution of algorithmic developments between research groups across different sites. This requires a versatile and efficient integration path for in-house developments into the platform, supporting arbitrary processing steps using different programming languages and input and output formats.

#### Data sovereignty.

Although the platform should enable joint projects within DKTK, it must put mechanisms into place that ensure full control over each site’s local data.

#### Data exploration.

Because algorithms are usually designed for specific types of input data, image properties such as modality, protocol, examined body parts, or patient characteristics are essential for selecting suitable training and test cases. The platform should enable users to filter appropriate data easily from existing image collections. The filters that identify a specific cohort should be shareable with partners.

#### Scalability.

As a service-oriented application, the platform should ensure a high level of scalability. For example, an increasing demand should be responded by spawning more instances of a service that is under high load.

#### Maintenance.

To ensure long-term sustainability of the platform, all platform instances in the consortium need to be kept up to date. Thus, to meet new developments and the continuous change in requirements, the platform must be easy to maintain, update, and expand. The installation and maintenance of each individual instance must be possible by nonspecialized technical staff of each site.

### Platform Architecture

**FIG 1.**
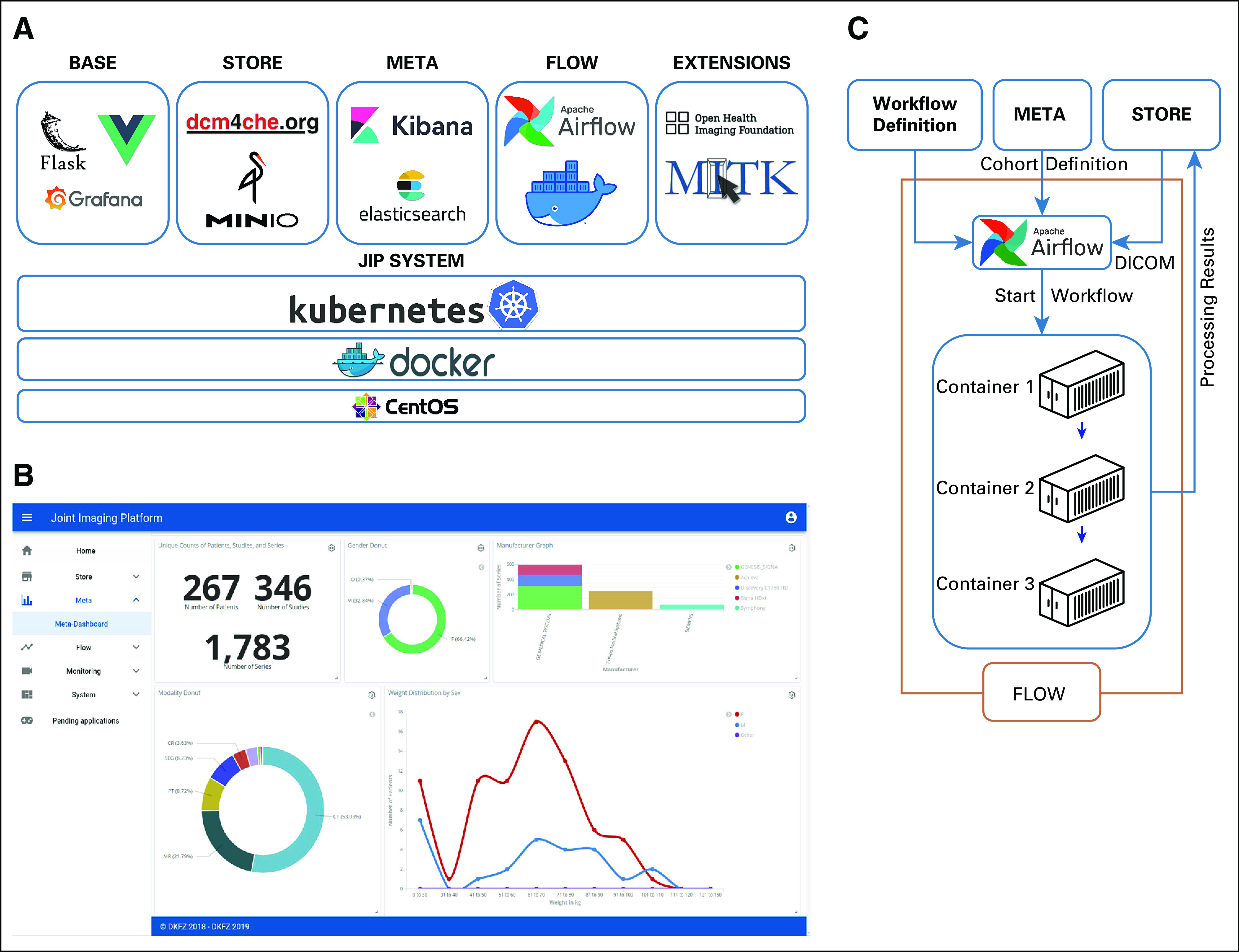
The Joint Imaging Platform (JIP) architecture and design principles. (A) Technology stack of the JIP: on the basis of any Linux-based operating system, the system components are realized as Docker containers and managed by Kubernetes. The actual application layer is structured in different functional units. (B) JIP BASE offers a common user interface, which is entirely Web based. The example shows the JIP META visualization of Digital Imaging and Communications in Medicine (DICOM) metadata in a Kibana dashboard. (C) Exemplary interplay between different JIP components: cohorts are defined in JIP META and handed over to JIP FLOW for data processing. Data input and output data are realized through communication with JIP STORE. (All trademarks and logos are the property of their respective owners.)

In response to these requirements, we designed a system that is structured into five building blocks (Fig 1). JIP SYSTEM realizes the technical basis and acts like an operating system of the platform. It takes care of provision, monitoring, and communication of services within the platform. JIP BASE consists of the more task-related components (eg, for the main user interface and authentication). JIP STORE contains components for data handling, management, and storage; JIP META contains components for metadata management, subject and image search, and selection; and JIP FLOW contains components for the controlled execution of processing sequences. Each of the functional units was realized based on open-source technologies. We have designed the system to be located within the protected hospital IT infrastructure. This allows for processing of the entire available patient data and facilitates integration into local procedures. The Data Supplement provides more detailed information about the individual components.

**FIG 2.**
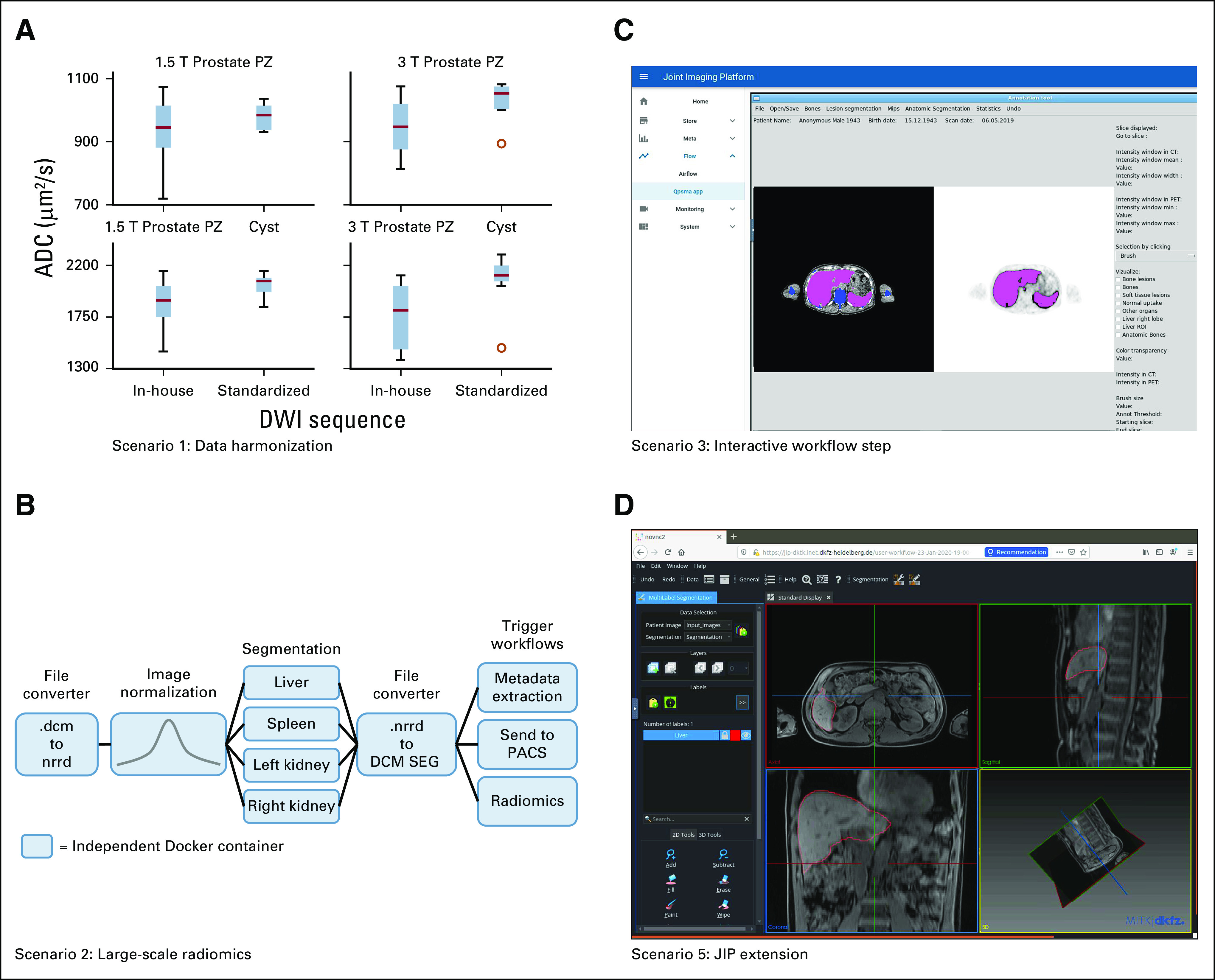
Scenarios realized within the Joint Imaging Platform (JIP). (A) Comparative intraindividual magnetic resonance imaging measurements of a traveling volunteer across sites. Apparent diffusion coefficient (ADC) values in the prostate were analyzed in the peripheral zone (PZ) (top row) as well as in the prostate cyst (bottom row), using 1.5 T (left) and 3 T (right). Sequence standardization led to substantially reduced variance in comparison with in-house (not standardized) sequences. (B) Exemplary organ segmentation workflow as realized in scenario 2. (C) Interactive workflow component of the qPSMA software^37a^ as realized in scenario 3. (D) Exemplary JIP extension offering a variety of tools for image segmentation. For this purpose, the Medical Imaging Interaction Toolkit (MITK) was wrapped in a Docker container and runs directly in the browser. DWI, diffusion-weighted imaging; PACS, picture archiving and communication system.

Because methods, technologies, and requirements in research are constantly evolving and the JIP is designed as an open platform for the community, extensibility of the platform for additional tools to explore, examine, or analyze medical data is crucial. Open interfaces, which are provided within the platform, allow a high degree of flexibility. This even extends to services that were originally not developed for use within a Web environment; the JIP offers Virtual Network Computing as an interface able to stream complete desktop applications to a Web browser. Some of the already integrated extensions (Fig 2D) are described in more detail in the Data Supplement. The seamless interaction of the JIP with other platform initiatives is also detailed in the Data Supplement.

## RESULTS

In this section, exemplary and complementary use cases that are realizable within the JIP and that cover all aspects of the previously defined platform requirements are described. The successful implementation of the JIP and its capabilities are further demonstrated in an overview of the current clinical and technical site involvements.

### Use Case 1: Data Harmonization and Integration

To enable comparability of study results, mutually agreeable scanner configurations or guidelines that achieve a more standardized imaging of certain patient groups are desirable in multicenter studies. The JIP supports such harmonization of data by enabling the development of algorithms that robustly handle multicenter data, including all the protocol and quality variations.

Within the DKTK consortium, the imaging protocols for diffusion-weighted magnetic resonance imaging of the brain and the prostate were standardized and validated using the JIP. The effect of the harmonization on the quantitative apparent diffusion coefficient measurements was demonstrated in comparison with canonical in-house sequences before and after standardization, exemplarily shown for prostate measurements (Fig 2A). Levene’s test revealed that there were significant differences between the variances before and after harmonization at 3 T (peripheral zone [PZ], *P* = .004; cyst, *P* = .00005) but not at 1.5 T (PZ, *P* = .072; cyst, *P* = .076).

### Use Case 2: Automatic Large-Scale Radiomics Analysis

In this scenario, a large number of images are processed using fully automatic image quantification algorithms, more specifically a shape model–based organ segmentation covering segmentations for kidneys, liver, and spleen in abdominal computed tomography (CT) scans^36^ followed by a radiomics analysis of the resulting objects.^37^

Complex workflows are realized in the JIP by concatenating individual processing steps and pipelines. The generated segmentations are pushed into JIP STORE and trigger the subsequent radiomics workflow, which automatically extracts the radiomics features from the provided organ masks. All metadata of the generated DICOM-SEGs are automatically extracted and pushed into JIP META. The combination of Kubernetes and Apache Airflow allows automatic parallel execution of each pipeline instance across the configured computing cluster, reducing computation time through transparent parallelization. Figure 2B illustrates the fully automated processing workflow, which is formally defined as a directed acyclic graph with Docker containers as nodes.

### Use Case 3: Interactive Analysis

This scenario demonstrates that interactive desktop applications can be shipped via containers and integrated into otherwise automatic JIP processing workflows. The desktop application qPSMA^37a^ for the interactive quantification of whole-body tumor volume of patients with prostate cancer using prostate-specific membrane antigen–positron emission tomography/CT images, developed by the Department of Nuclear Medicine at the Technical University Munich, was integrated into such a semiautomatic workflow. In this specific workflow, time-consuming preprocessing steps are automatically performed before the manual annotation step is triggered (Fig 2C). After the expert’s manual interaction, the automatic processing pipeline continues. No software needs to be installed on the annotators’ workstations, and multiple instances can be started simultaneously, also within a workflow, allowing parallel and more complex annotation workflows that might involve multiple users.

### Use Case 4: Federated Data Analysis

**FIG 3.**
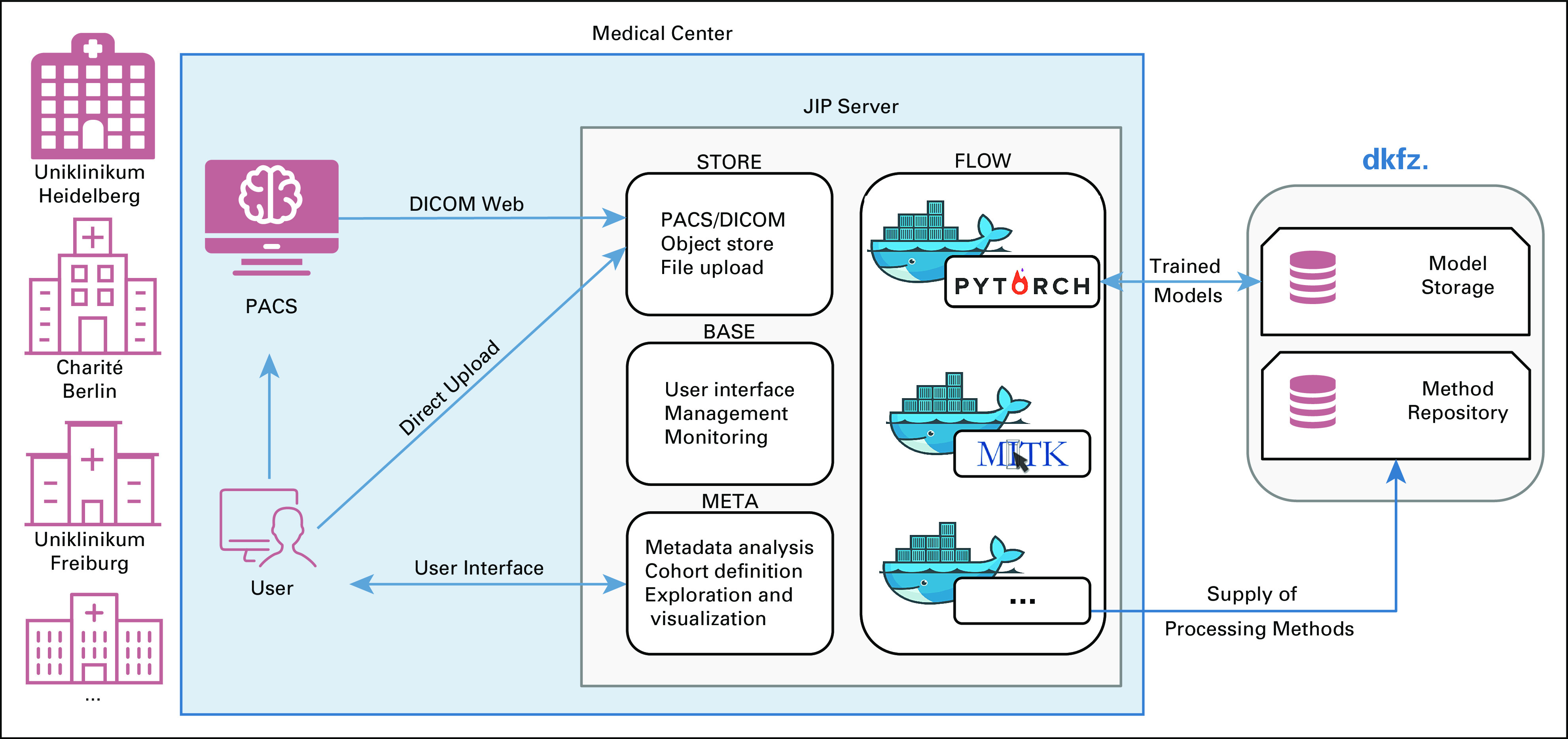
Clinical integration and federated setup of the Joint Imaging Platform (JIP). The JIP is located within the protected hospital information technology infrastructure, in principle allowing it to process the entire available patient data and facilitating its integration into local procedures. A central model storage was established at German Cancer Research Center to allow secure and access-controlled distribution and exchange of the platform itself, the analysis methods, and processing results in the form of containers. (All trademarks and logos are the property of their respective owners.) DICOM, Digital Imaging and Communications in Medicine; PACS, picture archiving and communication system.

This use case focuses on the cross-site distribution and application of analysis tools (Fig 3). As shown in the previous use cases, once a tool is packaged in a container, it can be executed on the platform independently of its grade of automation or interactivity level, building the foundation for a federated data analysis. The Helmholtz incubator project Trustworthy Federated Data Analysis,^38^ based on the JIP technology, is investigating trustworthy and regulatory compliant federated computing concepts. A hurdle in federated data analysis is the heterogeneity of data and metadata across sites, even when using standards such as DICOM. To approach this challenge, there is ongoing work to semiautomatically create mappings between different local conventions to a standardized metadata format. As a proof of concept, the development of this project will be validated in a federated radiation therapy study, aiming to reproduce results that were already generated and published by traditional means (ie, by pooling the data from all sites in the context of the DKTK Radiation Oncology Group study).^39^

### Site User Engagement and Projects

Recently, developers at local sites have started to become involved and to migrate their processing workflows into the JIP. Measures to further encourage community involvement include a detailed developer guide and documentation,^40^ together with an open-source release of the codebase.^41^ The first JIP tech workshop has recently taken place, and further events such as hackathons and workshops are planned.

Since the initial release, several projects have started to or plan to use the platform. A subset of projects is listed in Table 1. The spectrum of applications reaches from simple data management to complex application of modern ML algorithms. For example, the surgical ARMANI trial will use the platform to investigate imaging associated with different resection strategies of liver metastases. In the course of the project outlined in case 3, the lesion load in prostate cancer will be examined.

**TABLE 1.**
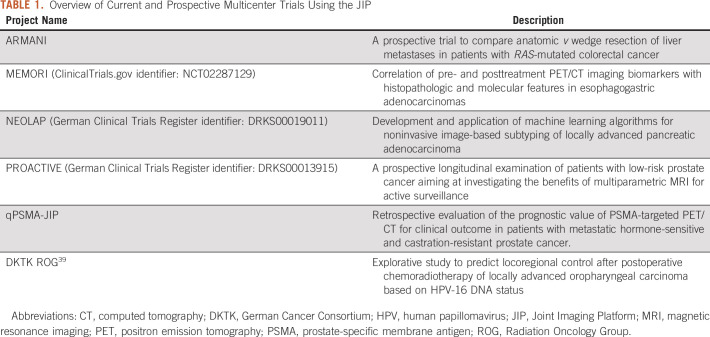
Overview of Current and Prospective Multicenter Trials Using the JIP

## DISCUSSION

The JIP provides a unified infrastructure across radiology and nuclear medicine departments of 10 university hospitals in Germany. The platform leverages state-of-the-art industry standards for cloud computing while adhering to on-premise hosting and execution. The technology stack is typical in modern cloud systems and can also easily be deployed using one of the leading commercial providers of cloud services.

The JIP offers standardized image processing by leveraging successfully established mechanisms from other fields. For example, we implemented a strictly browser-based interaction as in Galaxy^31^ and decentralized execution of algorithms as it was suggested by KETOS for textual data,^28^ while enabling the potential of container-based federated learning as suggested by the Personal Health Train.^32^

The decentralized approach enables compliance with data protection rules according to the European General Data Protection Regulation (GDPR) and the Health Insurance Portability and Accountability Act (HIPAA). We provided support in the handling of data protection questions to the sites by distributing technical and organizational measures as required by the recently introduced GDPR. The decentralized data storage facilitates additional GDPR requirements such as consent management (including the handling of consent withdrawal), data transparency, and the right to rectification and erasure. Depending on the study protocol, data pseudonymization and anonymization can be designed directly into the processing workflow, facilitating the duties of the data controller as required by the GDPR. These principles naturally extend to the requirements of the HIPAA, such as central processing steps for personal health information management.

Because of regional differences, site-specific data protection concepts often represent specific challenges. For example, some clinical IT networks do not grant permanent external communication. This complicates scenarios like methods exchange or federated learning by adding manual steps and preventing automated updates. In addition, the server must be installed and maintained independently at each site. This significantly increases the need for reliability and automation of maintenance routines. We have addressed this issue on several levels, from the basic system architecture up to documentation and support measures. The first release cycles have shown that our measures were successful.

The integration of electronic health records (EHRs) will be evaluated in upcoming versions. The contained information might serve as an important pretest probability for certain medical conditions and should be available as input for ML algorithms. Additional nonimaging parameters can, for instance, be taken into account by linking to the DKTK CCP-IT.^42,43^ Including radiologic reports from the radiologic information system presents an additional data source and allows for an even more powerful stratification of patient subgroups.

In the context of electronic case report forms and EHRs, the important aspect of data quality has been investigated extensively.^44-47^ With a similar intention but with a focus on imaging, future work on the JIP will include the development of automatic AI-based data quality assessment methods based on image metadata as well as the actual image data itself.

For a clinical application of AI-based methodologies, the trustworthiness of such techniques is also a key challenge. Trust can only be generated by a deep understanding of an algorithm's decision making, which can be promoted by new techniques of explainable AI, where the JIP as a research platform located in multiple university hospitals is the ideal tool to develop and test such approaches.^27^

To conclude, we have established a flexible decentralized analysis platform for medical images that respects data sovereignty and protects privacy by sharing algorithms instead of data. We observed that the availability of the JIP led to an unprecedented level of communication and collaboration within the radiologic and nuclear medicine research community of the DKTK. An increasing number of clinical studies have committed to use the JIP (Table 1). In addition, several requests for further extensions have been made (eg, supporting histopathology data).

Because DKTK is not the only collaborative network that is in need of a research imaging platform, the core implementation of the JIP will be available as an open-source software project named Kaapana.^48^ By providing the platform and source code, we hope to mitigate the compatibility gap between systems in the heterogeneous clinical IT landscapes and lay the foundation for unprecedented research opportunities in data-driven medicine.

## Figures and Tables

**TABLE A1.**
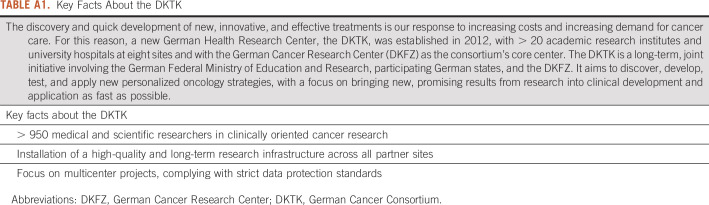
Key Facts About the DKTK
